# Periodontal conditions, low birth weight and preterm birth among postpartum mothers in two tertiary health facilities in Uganda

**DOI:** 10.1186/1472-6831-14-42

**Published:** 2014-04-28

**Authors:** Louis Muwazi, Charles Mugisha Rwenyonyi, Moses Nkamba, Annet Kutesa, Mike Kagawa, Godfrey Mugyenyi, Godfrey Kwizera, Isaac Okullo

**Affiliations:** 1 Department of Dentistry, School of Health Sciences, College of Health Sciences, Makerere University, Kampala, Uganda; 2 Department of Obstetrics and Gynecology, School of Medicine, College of Health Sciences, Makerere University, Kampala, Uganda; 3 Department of Obstetrics and Gynecology, Mbarara University of Science and Technology, Mbarara, Uganda; 4 Department of Dentistry, Mbarara University of Science and Technology, Mbarara, Uganda

**Keywords:** Preterm birth, Low birth weight, Periodontal disease

## Abstract

**Background:**

Literature reports have indicated an increase in research evidence suggesting association between periodontal disease and the risk of pre-term birth (PTB) and low birth weight (LBW). Periodontal diseases in Uganda have been documented as a public health problem, but their association to adverse pregnancy outcomes is unknown. This study was conducted to assess the association between periodontital diseases in postpartum mothers and PTB and LBW of babies in Mulago and Mbarara referral hospitals.

**Methods:**

This was a cross sectional study using medical records, clinical examination and oral interview of mothers at the two tertiary health facilities. Mothers with singleton babies from Mulago (n = 300) and Mbarara Hospital (n = 100) were recruited for the study. The women were clinically examined for periodontal disease by 2 trained and calibrated dentists. Data on PTB and LBW were retrieved from medical records. The data were analyzed to determine the relationship between the four parameters for periodontal disease (bleeding gingiva, periodontal pockets, gingival recession and calculus with plaque deposits) and the adverse pregnancy outcomes. Frequency distribution was used to describe the data. Bivariate and multivariate analyses were used to study the association between the periodontal diseases and adverse pregnancy outcomes.

**Results:**

Approximately 26% and 29% of the postpartum mothers examined had bleeding gingiva and periodontal pockets of 4 mm or more deep, respectively. Advanced periodontitis i.e. pocket depth ≥ 6 mm was recorded in 13 (3.6%) of the mothers. Calculus with plaque deposits were recorded in 86% (n = 343) of the mothers. Gingival recession was recorded in 9.0% of the mothers and significantly and directly related to birth weight (p < 0.05).

**Conclusion:**

Periodontal conditions of postpartum mothers in this study were found to be better than previously reported amongst the Ugandan population. Bivariate analysis showed a significant association only between gingival recession and low birth weight. However, this finding should be interpreted with caution as it could have occurred by chance.

## Background

Periodontal diseases are a group of oral inflammatory diseases caused by bacterial plaque and influenced by host response factors [1]. There are two main types of conditions; gingivitis which is inflammation of soft tissues surrounding the tooth (the gingiva), and periodontitis involving apical migration of the periodontal ligament attachment and destruction of the connective tissue and alveolar bone that support the teeth [2,3].

Some of the important clinical features include alterations in the gingival color, pocket depth, position of the epithelial attachment and tendency to bleeding [4]. In most developed countries, pregnancies are planned, complications are few and outcomes are generally favorable for both mother and infant, however, adverse outcomes are far more frequent in the developing world [5]. Much of the published research in the area of adverse pregnancy outcomes, are based on proxy outcomes for mortality and severe morbidity. The most commonly studied of these proxies being low birth weight (LBW) and its constituents, preterm birth and intrauterine growth retardation (IUGR) [5].

Namiro et al. also found that preterm birth (PTB) and low birth weight (LBW) are more prevalent in developing than developed countries [6]. South Asia and Sub-Saharan Africa account for almost two-thirds of the world’s preterm babies [7]. In a previous survey in Uganda, one in seven newborn babies had LBW and required extra care to survive and thrive [8]. Low birth weight, i.e. birth weight of <2.5 kg, remains a significant public health problem in many parts of the world and is associated with a range of both short- and long-term adverse effects [9]. Systemic maternal infections are hypothesized to raise the risk of placental infection, premature rupture of membranes, premature labor and preterm birth by release of inflammatory cytokines and increased prostaglandin production [10]. Periodontal disease as a low grade chronic infection for example can be a challenge at systemic level due to the large epithelial surface that could be ulcerated in the periodontal pockets [11].

Although a number of studies have shown that bacterial vaginosis is related to preterm birth and low birth weight, which in effect is a significant cause of morbidity and mortality, it is also possible that other infectious processes including periodontal disease contribute to PT/LWB [12].

Indeed recent years have witnessed an increase in research evidence suggesting associations between periodontal disease and an increase in systemic disease [13]. After the publication of results by Offenbacher and co-authors in 1996 [14] from a case controlled study suggesting that women who delivered PT/LBW infants had poorer periodontal health than mothers with normal birth weight, a number of studies have come up trying to validate this observation [12]. The majority of the studies, especially those carried out in economically disadvantaged populations; suggest that periodontal disease is associated with increased risk of various adverse pregnancy outcomes such as preterm birth and low birth weight [13].

Clinical trials at the Center for Oral and Systemic Disease at the University of North Carolina, reported the presence of higher levels of *Porphyromonas gingivalis*, *Bacteroides forsythus*, *Actinobacillus actinomycetemcomitans* and *Treponema denticola*, organisms normally associated with periodontal disease, in mothers of PTB and LBW babies as compared to normal controls [14].

Opinion seemed to defer early when investigators like McGaw (2002) suggested that prospective and eventually interventional studies are necessary before periodontitis can be considered as a causal factor for PLBW [15]. In a meta-analysis, Khader et al., suggested that appropriate evidence was not available from good-quality observational research that could support the association between periodontal disease and PTB/PLBW [16].

Some intervention studies suggest a reduction in the risks of preterm birth and preterm low birth weight after mechanical periodontal therapy during pregnancy. López et al. found a reduction in the rate of preterm births and/or low birth weight in women that received periodontal treatment before the 28th gestation week when they were compared with women that did not receive any treatment. This reduction was significant for periodontally healthy women compared with women with gingivitis [17], and with periodontitis [18].

In a systematic review by Scannapieco et al. [12], where they selected 12 cases that they thought were high quality research out of the identified 660 studies, their conclusion was suggestive of periodontal disease being a risk factor for PT and LBW although they were cautious and recommended further longitudinal, epidemiological and interventional studies.

Another systematic review published in 2005 by Xiong et al. analyzed the results of 25 articles. The majority, 18 out of the 25 studies, suggested an association between periodontal disease and increased risk of adverse pregnancy outcomes [13]. Paquette (2006), however, noted that in spite of the building evidence supporting an association between periodontal disease and adverse pregnancy outcomes; there remains some potential bias due to inconsistencies in the definition of periodontal disease and the relatively limited number of randomized controlled trial studies [19].

On the other hand, some clinical and laboratory studies have shown that intrauterine infections are highly prevalent among women who give birth prematurely [20]. Although the ascending infection from the vagina is considered the most common route of infection, they also suggest a hematogenous spread of organisms from other body sites to the uterus as a second route. Indeed organisms with an oral origin, such as *Fusobacterium nucleatum* and *Capnocytophaga* spp have been associated with intrauterine infections; *F. nucleatum* being one of the most frequently isolated microorganism from the infected uterus [21,22].

In periodontal disease, transient bacteremia may occur probably leading to selective colonization of undesired sites [23]. Studies by Han et al. have shown that hematogenous injection of orally related *F. nucleatum* resulted in preferential localization to placental blood vessels from which it crossed the endothelium to the amniotic fluid and induced premature delivery, and stillbirths in a pattern similar to that observed in humans. This finding supports the hypothesis that after transient bacteremia, oral *F. nucleatum* translocates to the pregnant uterus and possibly to the fetus hematogenously [24]. Observations by Jiang et al. [25] showed that several large clinical randomized controlled trials failed to demonstrate that periodontal therapy during pregnancy reduces the incidence of adverse pregnancy and birth outcomes. They proposed a new approach of using pre-conception treatment of periodontal disease to improve periodontal status during late pregnancy and subsequent birth outcomes: this may throw more light on the debate.

Uganda has a big economically disadvantaged population and, periodontal diseases have been documented as a public health problem especially in the rural communities with a prevalence of 62% in adults [26]. Coincidentally, there is also a high neonatal mortality in Uganda usually associated with preterm birth and low birth weight [8]. However, the contribution of periodontal diseases to these pregnancy outcomes is still unknown. The aim of the study was to determine the magnitude of periodontal diseases and their association to preterm births and low birth weight in postpartum mothers in Mbarara and Mulago referral hospitals.

## Methods

### Study setting

This was a cross sectional study conducted in maternity units in Mulago and Mbarara hospitals between May and November 2012. The two health facilities are national referral and teaching hospitals in Uganda. Mulago Hospital has a capacity of 1,500 beds and is located in Kampala, the capital city of Uganda. Mbarara Hospital has a 300 bed capacity and is about 280 km west of Kampala. Mulago and Mbarara hospitals attend to approximately 80 and 40 deliveries per day, respectively.

### The study population

The study population included postpartum mothers aged 18 years or above who delivered singleton babies within one or two days prior to recruitment into the study in both hospitals. The women were selected consecutively from the maternity units. Mothers of infants with severe congenital malformations, systemic conditions such as HIV infection, uncontrolled diabetes mellitus, or any medical condition requiring chronic medication and those without teeth in one or more sextants were excluded. Based on proportionate sampling, 300 and 100 mothers were recruited from Mulago and Mbarara hospitals, respectively.

### Clinical examination

One to two days after delivery, the mothers were clinically examined for their periodontal diseases in their hospital room by two trained and calibrated dentists. The examiners were blinded to the physical condition of the baby to minimize bias. We defined periodontal disease by a combination of the following four parameters: 1) Probing pocket depth (PPD), 2) Bleeding on probing (BOP), 3) Calculus with plaque deposits (CD) and 4) Gingival recession (GR) using the modified Community Periodontal Index (CPI) with the following criteria [27].

0. No periodontal disease

1. Bleeding on probing

2. Calculus with plaque seen or felt by probing

3. Pathological pocket 4 – 5 mm

4. Pathological pocket 6 mm or more

5. Gingival recession

We recorded pocket probing depth and gingival recession using a World Health Organization recommended Community Periodontal probe (Hv-Friedy, Chicago, IL, USA), at six sites per tooth on the 6 index teeth (16, 11, 26, 36, 31 and 46) translating into 36 sites per participant. If the index tooth was not present, the next tooth was examined. Probing pocket depth was measured in mm as the distance from the gingival margin to the bottom of the pocket [28].

### Medical information

Medical history was obtained through oral interview after the oral examination to get background information on maternal age, nationality, educational level, marital status, and employment during pregnancy, tobacco smoking before and during pregnancy, and number of prenatal visits. Other information included: birth weight of the baby, parity, infection, complications during pregnancy, intra-uterine growth retardation (IUGR), preterm premature rapture of the membranes (PPROM), maternal hemorrhage and onset of labor. Induced preterm birth included both induction of labor and caesarean section before labor.

### Ethical considerations

All procedures conformed to the protocol approved by Makerere University, School of Health Sciences Institutional Review Board and the respective hospital research and ethics committees. Informed consent was obtained from the participating mothers in accordance with Helsinki Declaration [29]. No personal identifiers were used in data collection and the information was kept under lock and key.

### Quality control

The data were double checked for errors and completeness at the end of each day’s work. Assessment of periodontal diseases was standardized through training of the two examiners by an experienced clinician (ML) on 20 patients in Makerere University Dental clinic before the start of the study. The inter-class correlation coefficients [30] were used to check for consistency in recording periodontal parameters. The correlation coefficients for probing depth were 0.83; calculus with plaque, 0.86 and gingival recession 0.87. During the main survey, reproducibility test of the periodontal disease recordings through blind duplicate examination in 10% (n = 40) of randomly selected mothers was done a day after the main examination. The correlation coefficient in recording probing depth was 0.86; calculus with plaque deposits, 0.89, and gingival recession, 0.91.

### Data analyses

The data were double checked for errors and completeness and entered into a computer for analyses using Statistical Package for Social Sciences Inc. (SPSS, version 17 for windows, Chicago, Illinois, USA). The frequency distribution was used to describe the data. The infant’s birth weight was categorized as 0 = <2.5 kg and 1 = ≥2.5 kg. The gestation age was categorized as 0 = 28–36 weeks and 1 = ≥37 weeks. The periodontal diseases (bleeding gingiva, pocket depth of ≥4 mm and ≥6 mm, calculus with plaque deposits, and gingival recession) were individually categorized as 0 = no, and 1 = yes. The demographic and obstetric factors were also categorized (Table 1 and Table 2). Spearman’s rank correlation coefficients were used to study the bivariate association between quantitative variables. Partial correlation analyses were used to assess any association between periodontal diseases and adverse pregnancy outcomes. Binary logistic regression analysis was used to assess any risk indicators of adverse pregnancy outcomes. The inter-class correlation coefficients were used to assess any systematic errors in recording the periodontal diseases. The probability of significance was set at the two sided 5% level.

**Table 1:** The frequency distribution of the postpartum mothers according to demographic factors (n = 400)

**Categories**	**Categories**	**Number**	**Percent**
Age	18-20	110	27.5
	21-25	139	34.8
	26-30	102	25.5
	31-45	49	12.2
Marital status	Married	283	70.8
	Cohabiting	61	15.3
	Single	50	12.5
	Separated	6	1.5
Educational level (n = 381):	Primary	153	38.3
	Secondary	194	48.5
	Tertiary	34	8.5
Employment	Business	91	22.8
	Civil servant	31	10.3
	Others	209	52.3
Parity (n = 361):	Nullparous	135	37.4
	Multiparous	226	62.6
Type of delivery	Vaginal	244	61.0
	Ceasarian	156	39.0
ANC attendance (n = 397)	1-3 times	196	49.4
	≥4 times	201	50.6
Vaginal bleeding during pregnancy	Yes	25	6.3
	No	375	93.7

**Table 2:** The frequency distribution of postpartum mothers according to obstetric factors (n = 400)

**Variable**	**Categories**	**Number**	**Percent**
Illness during pregnancy:	Yes	126	31.5
	No	274	68.5
Medication during pregnancy (n = 126)	Yes	122	96.8
	No	4	3.2
Class of medicine taken (n = 122)	Antibiotics	52	2.6
	Antimalarials	54	44.3
	Pain killers	9	7.4
	Antifungal (vaginal pessaries)	7	5.7
ANC attendance	Yes	397	99.3
	No	3	0.7
No. of ANC attendance (n = 397)	1-3	196	49.4
	≥ 4	201	50.6
Height of fundus (n = 359)	20-36 cm	29	8.1
	≥37 cm	330	91.9
Estimated age of gestation at delivery (n = 374)	28-36 weeks	194	48.5
	≥37 weeks	206	57.5
Infant’s weight at delivery (n = 392)	< 2.5 kg	191	47.8
	≥ 2.5 kg	206	51.5
Parity (n = 361):	Nullparous	135	37.4
	Multiparous	226	62.6
Type of delivery	Vaginal	244	61.0
	Ceasarian	156	39.0
Vaginal bleeding during pregnancy	Yes	25	6.3
	No	375	93.7

ANC – antenatal clinic.

## Results

Overall, 62.3% (n = 249) of the postpartum mothers were ≤ 25 years (Table 1) with no significant difference in the distribution between Mulago and Mbarara Hospital (p > 0.05), hence the data were pooled. Most mothers (70.8%, n = 283) were married and approximately half (48.5%) had attained secondary school education (Table 1).

Most mothers were at para 2 and 3,and had unspecified employment (Table 1). Most of the mothers were taking antimalarial prophylaxis, while only one mother reported having used tobacco products, and the majority of them did not report illness during pregnancy (Table 2). Almost all the mothers (99.3%) had attended antenatal clinic with about half of them (50.6%) at least four times (Table 2). Fundal height of 37 cm and more was registered in 91.9% of the mothers and 86.6% had a gestation age of 37 weeks or more at delivery (Table 2), with about 3.8% of the mothers experiencing rupture of uterine membranes before the onset of labor. About 95% of the mothers had spontaneous labor onset with 61% having vaginal delivery (Table 2). Twelve (3%) of the mothers experienced vaginal bleeding within the week preceding delivery (Table 2). Thirty four (8.7%) of the infants had a birth weight of less than 2.5 kg (WHO definition of low birth weight).

Generally, tooth 16/17 and 26/27 had more prevalence of periodontal diseases (CPI score 1–5) than the rest of the teeth. On the other hand the upper central incisor (#11) had the lowest prevalence of periodontal diseases as compared to the rest of the teeth. Approximately 26% and 29.4% of the mothers had bleeding gingiva and periodontal pockets of 4–5 mm deep, respectively (Table 3 and Table 4). Gingival bleeding and periodontal pocket depth of 4–5 mm were not significantly associated with birth weight and gestation age (p > 0.05). Advanced periodontitis, i.e. pocket depth ≥ 6 mm was recorded in 3.6% of the postpartum mothers (Table 3 and Table 4). Calculus with plaque deposits were recorded in 86.0% of the mothers with neither significant association with birth weight of the infant nor the gestation age (p > 0.05). Gingival recession was recorded in 9.0% of the postpartum mothers and was significantly and directly related to birth weight (p < 0.017, Table 3), but not to gestation age (p > 0.05, Table 4). Based on partial correlation analysis between gestation age and periodontal pockets while controlling for gingival bleeding and gingival recession, there was no significant correlation (p > 0.05, t test). There was also no significant correlation in partial correlation analysis between infants’ birth weight and periodontal pocket depth while controlling for gingival bleeding (p > 0.05, t test). Furthermore, based on bivariate analysis, there was no significant association between adverse pregnancy outcomes and other quantitative variables (p > 0.05, t test), except between gingival recession, employment status and birth weight of the infant; features of chorioamnionitis, and birth weight of the infant (<0.05, Table 5). When the periodontal diseases, demographic and obstetric factors were assessed as risk indicators of adverse pregnancy outcomes, none of the variables entered into the logistic regression model.

**Table 3:** The frequency distribution of postpartum mothers according to their periodontal conditions and birth weight of their infants (n = 400)

**Periodontal condition**	**Categories**	**Birth weight (kg)**		**P-value**
		**< 2.5**	**≥ 2.5**	
Bleeding gum	Yes	50 (12.5)	54 (13.5)	0.961
	No	141 (35.4)	154 (38.6)	
Pocket depth 4–5 mm	Yes	44 (11.0)	59 (14.8)	0.224
	No	147 (36.8)	149 (37.4)	
Pocket depth ≥ 6 mm	Yes	6 (1.6)	8 (2.0)	0.490
	No	186 (46.6)	200 (50.1)	
Calculus with plaque deposits	Yes	163 (40.9)	180 (45.1)	0.823
	No	28 (7.0)	29 (7.2)	
Gingival recession	Yes	10 (2.5)	26 (6.6)	0.017
	No	181 (45.4)	183 (45.9)	

**Table 4:** The frequency distribution of postpartum mothers according to their periodontal conditions and gestation age (n = 400)

**Periodontal condition**	**Categories**	**Gestation age (weeks)**		**P-value**
		**28 - 36**	**≥ 37**	
Bleeding gum	Yes	51 (12.8)	54 (13.5)	0.921
	No	143 (35.8)	152 (38.0)	
Pocket depth 4–5 mm	Yes	47 (11.8)	57 (14.3)	0.350
	No	148 (37.0)	148 (37.0)	
Pocket depth ≥ 6 mm	Yes	5 (1.3)	9 (2.3)	0.980
	No	189 (47.3)	197 (49.3)	
Calculus with plaque deposits	Yes	166 (41.5)	178 (44.5)	0.698
	No	29 (7.3)	29 (7.3)	
Gingival recession	Yes	14 (3.5)	22 (5.5)	0.155
	No	181 (45.3)	183 (45.8)	

**Table 5:** **The Spearman’s rank correlation coefficients (r**^**s**^**) of gestation age and infant’s birth weight with periodontal conditions of postpartum mothers and their demographic, and obstetric factors (n = 400)**

**Periodontal condition**	**Gestation age**		**Birth weight of infant**	
	**r**^**s**^	**p-value**	**r**^**s**^	**p-value**
Bleeding gum	-0.005	0.921	-0.002	0.961
Pocket depth of 4–5 mm	0.047	0.352	0.061	0.225
Pocket depth ≥ 6 mm	0.037	0.457	0.035	0.491
Calculus	0.019	0.699	0.011	0.823
Gingival recession	0.071	0.156	0.120*	0.017
Demographic variables				
Age of the mother	0.065	0.383	0.045	0.123
Level of education	0.031	0.546	0.022	0.668
Employment	0.012	0.061	0.0115*	0.034
Marital status	0.037	0.464	0.063	0.210
Parity	0.001	0.988	-0.015	0.774
Type of delivery	-0.045	0.375	-0.026	0.608
Obstetric factors				
No. of ANC attendance	0.084	0.092	0.025	0.617
Aneamia	0.004	0.932	0.006	0,898
Labour onset	-0.017	0.740	-0.021	0.673
Vaginal bleeding	0.039	0.440	0.022	0.661
Medication	0.009	0.857	0.008	0.872
Repture of membranes	0.072	0.152	0.075	0.136
Features of chorioamnionitis	0.123*	0.014	0.125*	0.012

*Spearman’s rank correlation coefficients that are statistically significant.

## Discussion

This was a pilot study based on a sample of postpartum mothers (n = 400) in two tertiary health facilities in Uganda. This study shows a significant association between gingival recession and low birth weight, indicating that periodontal disease maybe a factor influencing pregnancy outcomes in Ugandan women. However, there is a 50% probability of getting this positive finding just by the play of chance. Never the less, many of the studies carried out in economically disadvantaged populations suggest that periodontal disease is associated with increased risk of adverse pregnancy outcomes such as preterm birth and low birth weight [6,13].

It should be noted that mothers who had still births were excluded in the present study due to fear of further traumatizing them. This exclusion may have led to loss of data resulting into inadequate statistical power and imprecise results. The examiners were calibrated and the reliability test in recording periodontal diseases gave an almost perfect agreement [31], a further indication of the consistency of the findings of this study with previous studies. They were further blinded to the preterm/at term status of the births which ruled out any possible misclassification due to bias. Most mothers were young, which could explain the low prevalence of periodontal disease (38.8%) amongst this population. This is in agreement with studies by Pitiphat et al. [11] who noted in their study that women who reported having periodontitis were in an older age group. Other reports also indicated that periodontal disease is more prevalent in advanced age [32].

Over all, in the present study, about 26% and 29.4% of the mothers had gingival bleeding and periodontal pocket depth of 4 mm or more, respectively (Table 3). Gingival bleeding on probing recorded among the mothers in this study could be suggestive of gingivitis and possibly transient bacteremia that have been hypothesized to lead to adverse pregnancy outcomes. This has previously been reported in other studies where chronically injured and inflamed oral mucosa have been associated with increased quantities of periodontal pathogens dramatically increasing transient bacteremia in pregnancy [23,33].

In the present study, about 9 out of 10 mothers had a fundal height of 37 cm and more, which corresponds to the reported gestation age of pregnancy before delivery (Table 2). This could probably be attributed to the high (99.3%) antenatal attendance recorded among the postpartum mothers (Table 3).

## Conclusion

Generally, the periodontal conditions of these postpartum mothers were found to be better than previously reported amongst the Ugandan population. Bivariate analysis showed a significant association between gingival recession and low birth weight, however, no other parameters of periodontal disease were significantly related to adverse pregnancy outcomes. This finding should therefore be interpreted with caution as it could have occurred by chance.

It is recommended that a large sample size be used to confirm whether the lack of association between other parameters of periodontal conditions and adverse pregnancy outcomes was real.

## Competing interests

The authors declare that there are no competing interests.

## Authors’ contributions

LM, CMR, IO contributed to the design of the study. MN, AK performed the clinical examinations of postpartum mothers. GK, MK, GM did the analysis of the data. All authors contributed to the preparation of the manuscript and approval of the final version.

## Pre-publication history

The pre-publication history for this paper can be accessed here:

http://www.biomedcentral.com/1472-6831/14/42/prepub
